# Viral and bacterial microorganisms in Vietnamese children with severe and non-severe pneumonia

**DOI:** 10.1038/s41598-023-50657-5

**Published:** 2024-01-02

**Authors:** Xuan Duong Tran, Van-Thuan Hoang, Ndiaw Goumballa, Thi Nguyet Vu, Trong Kiem Tran, Thi Dung Pham, Thi-Loi Dao, Thi Thuy Vu, Duy Cuong Nguyen, Quoc Tien Nguyen, Pierre Marty, Philippe Gautret

**Affiliations:** 1 Thai Binh University of Medicine and Pharmacy, Thai Binh, Vietnam; 2 Institut Hospitalo-Universitaire (IHU)-Méditerranée Infection, 19-21 Boulevard Jean Moulin, 13385 Marseille Cedex 05, France; 3 IRD, AP-HM, SSA, VITROME, Aix Marseille University, Marseille, France; 4 Thai Binh Paediatric Hospital, Thai Binh, Vietnam; 5 Inserm, C3M, Université Côte d’Azur, Nice Cedex 3, France; 6 Parasitologie-Mycologie, Centre Hospitalier Universitaire l’Archet, Nice Cedex 3, France

**Keywords:** Microbiology, Molecular biology, Diseases

## Abstract

To investigate potential respiratory pathogens in children with community-acquired pneumonia (CAP) and risk factors for severe disease. This prospective study was conducted among 467 children at the Thai Binh Paediatric Hospital, Vietnam between 1 July 2020 and 30 June 2021. Clinical data and laboratory results were collected. Twenty-four respiratory microorganisms were tested from nasopharyngeal swabs using real-time PCR. Logistical regression was used to estimate a factor’s adjusted odd ratios of the severity of disease. Mean age of patients = 15.4 ± 13.3 months, 63.0% were male. Over 97% of patients had a positive PCR result. 87% of patients were positive for multiple (up to eight) microorganisms. Rhinovirus (46%), respiratory syncytial virus (RSV) (24%), enterovirus (17%), and parainfluenza viruses-3 (13%) were the most frequent viruses. *H. influenzae* (61%), *S. pneumoniae* (45%) and *M. catarrhalis* (30%) were the most common bacteria. 128 (27%) cases were classified as severe pneumonia. Presence of smokers at home (aOR 2.11, 95% CI 1.27–3.52, *P* value = 0.004), CRP level ≥ 50 mg/dL (aOR  6.11, 95% CI  3.86–9.68, *P* value < 0.0001), RSV (aOR  1.78, 95% CI  1.07–2.96, *P* value = 0.03) and *H. influenzae* (aOR  1.66, 95% CI  1.03–2.67, *P* value = 0.04) PCR detection associated with a higher risk of severe pneumonia; ,. Causative agents of pneumonia in children are complex. Children positive with RSV and *H. influenzae* need to be closely monitored to prevent severe pneumonia.

## Introduction

Community-acquired pneumonia (CAP) remains a leading cause of morbidity and mortality among infants and children worldwide, particularly in resource-limited settings^1^. CAP imposes a significant burden on healthcare systems, contributing to hospital admissions and posing challenges in diagnosis and management. The identification of the aetiological agents responsible for CAP is crucial for effective patient care, antimicrobial stewardship, and the development of preventive strategies^2^. While CAP has a similar incidence rate in developed and developing countries, the mortality rate is higher in low-income countries^3^. Pneumonia remains the leading killer of children under the age of five, and is responsible for approximately 20% of all deaths, with 70% of these deaths occurring in southern regions of Asia^4^. Respiratory pathogens responsible for respiratory tract infections (RTIs) vary from region to region, possibly owing to climate, culture, and geographical differences. In addition, clinical features cannot be used to distinguish between viral infections and bacterial infections. In low-income countries or in poor settings, antibiotics are frequently prescribed due to concerns over bacterial systemic infections when faced with non-specific symptoms^5^.

In Vietnam, as in many other developing countries, CAP represents a major public health concern. The prevalence and diversity of pathogens associated with CAP among infants and children in Vietnam has been the subject of interest and ongoing investigation^6,7^. Traditional diagnostic approaches, including culture and antigen detection methods, have limitations in terms of accurately identifying the diverse array of pathogens responsible for CAP. To address this gap, molecular methods have emerged as powerful tools for pathogen detection due to their sensitivity, specificity, and ability to simultaneously screen for multiple organisms. In Thai Binh Paediatric Hospital, in northern Vietnam, 81% of all children who were admitted received antibiotic therapy, while infectious diseases accounted for 61% of cases^5^. Microbiological molecular methods now allow for the detection of respiratory pathogens at an earlier stage and with greater accuracy, reducing unnecessary antibiotics and improving patient management. This study aimed to describe the clinical characteristics, epidemiology, and potential respiratory pathogens using PCR in children with CAP in a Vietnamese hospital and to investigate the risk factors for severe disease.

## Materials and methods

### Study design and location

This prospective study was conducted between 1 July 2020 and 30 June 2021 at the Thai Binh Paediatric Hospital, a tertiary hospital in northern Vietnam with about 2000 hospitalised patients and 10 000 consultations per month^5^.

### Inclusion criteria

Patients aged five years old or under, who were hospitalised in the respiratory medicine ward with community-acquired pneumonia were enrolled in this study. Children over the age of five, as well as HIV-positive, immunocompromised and neutropenic patients, patients presenting with travel-related fever, or those with pneumonia occurring after 48 h of hospitalisation were excluded from this study.

### Data collection

Demographic information, vaccination status (relevant to respiratory tract infections), environmental conditions at home, epidemiological and clinical features, treatment and outcome were collected using a standardised questionnaire under the supervision of a physician. Children were considered to lacking immunisation if they had not received full vaccines, according to the expanded vaccination programme in Vietnam^8^. The tuberculosis vaccine is recommended as soon as possible, within 30 days of birth. Three doses of pentavalent vaccine (diphtheria, tetanus, pertussis, hepatitis B, and *Haemophilus influenzae* type B) and oral polio vaccine are recommended at 2–4 months. The measles vaccine is recommended at 9–11 months. Vaccines against influenza and invasive pneumococcal diseases are optionally available, however they are not free of charge, unlike those recommended by the expanded programme and are expensive.

Clinical and radiological data, routine laboratory results and therapeutic data were collected from medical files. Blood specimens were collected from all patients for white blood cell (WBC) counts and C-reactive protein (CRP) detection. Influenza A and B were detected in nasopharyngeal swabs using a rapid test (ASAN Easy Test Influenza A/B, ASAN Pharmaceutical CO., LTD, Korea).

A blood culture was performed in patients with a suspicion of sepsis. A sample of 2 mL–5 mL of venous blood was collected and inoculated into 20 mL culture medium (brain heart infusion broth (Oxoid, Basingstoke, UK) plus 0.05% sodium polyanethol sulfonate (Sigma-Aldrich, St. Louis, MO, USA). The vented bottles were incubated at 37 °C for up to seven days. Bottles were routinely sub-cultured onto solid media after 24 h and seven days, with additional subculture if turbidity was noted upon daily inspection. Bacterial isolates from blood were identified using the VITEK Mass Spectrometry System (Vitek MS; bioMérieux, Durham, NC, USA)^9^.

### Indicators of clinical severity

Pneumonia was diagnosed when infants and children presented with a cough and/or difficulty breathing and/or fever and tachypnoea or chest indrawing (retraction), and an abnormal lung examination^2,10^. The severity of pneumonia was assessed according to the guidelines of Pediatric Infectious Diseases Society and the Infectious Diseases Society of America and the British Thoracic Society^2,10^, as presented in Supplementary Table 1.

### Sample collection and PCR procedure

Nasopharyngeal swabs were collected for each patient and then transferred to Sigma-Transwab^®^ medium by a nurse in a standardised way. The samples were kept at room temperature before transfer to the laboratory department within 1 h and stored at −80 °C. The samples were then transferred to Marseille at −20 °C on dry ice before subsequent investigation.

Thermo Scientific KingFisher Flex extracted DNA and RNA from nasopharyngeal samples using the Kit NucleoMag^®^ Dx Pathogen (Macherey-Nagel, Germany) according to the manufacturer's instructions. The extracted products were tested by RT-PCR for common human coronaviruses (HCoV) and human parainfluenza viruses (HPIV) by one-step duplex quantitative RT-PCR amplifications of the HCoV/HPIV-R Gene Kit (Ref: 71-045, BioMérieux, Marcy l’Etoile, France), according to the manufacturer's recommendations. Each sample positive for HCoV was further tested by RT-PCR for coronavirus 229E, HK01, NL63 and OC43, as previously described^11^. Each sample which was positive for HPIV was further tested by RT-PCR for PIV types 1, 2, 3, and 4^12^. The Multiplex RNA Virus Master Kit (Roche Diagnostics, France) was used to detect influenza A, influenza B, human rhinovirus, human enterovirus, metapneumovirus, respiratory syncytial virus, SARS-CoV-2, and adenovirus by one-step simplex real-time quantitative RT-PCR amplifications. Real-time PCR amplifications were carried out using LightCycler^®^ 480 Probes Master kit (Roche Diagnostics, France) according to the manufacturer’s recommendations. The *SHD* gene of *H. influenzae*, the *phoE* gene of *Klebsiella pneumoniae*, the *nucA* and *amydo* genes of *Staphylococcus aureus*, the *lytA CDC* gene of *Streptococcus pneumoniae*, the *P1* gene of *Mycoplasma pneumoniae*, the *ctrA* gene of *Neisseria meningitides*, the *copB* gene of *Moraxella catarrhalis* and the *Toxin* gene of *Bordetella pertussis* were amplified as previously described^13,14^.

Negative controls (PCR mix) and positive controls (DNA or RNA for bacterial or viral strain) were added to validate each RT-PCR test. The results were treated by CFX manager Software version 3.1 following the manufacturer’s recommendations. A sample was considered to be positive when a cyclic threshold value was equal to or less than 35.

### Statistical analysis

Stata software was used for statistical analysis. Categorical variables were presented as percentages, while continuous variables were presented as median and interquartile. Our main outcome was the severity of community-acquired pneumonia among infants and children. Unadjusted associations between multiple independent factors and severe pneumonia were calculated. The results were presented by odds ratio (OR) with a 95% confidence interval (95%CI). Variables with *P* values < 0.2 in the univariate analysis were introduced in the multivariate model. Logistical regression was used to estimate a factor’s adjusted odd ratios of the outcome. A *P* value < 0.05 was considered to be statistically significant.

### Ethics statement

The study was accepted by the Thai Binh University of Medicine and Pharmacy institutional review board (No. 498/HDDD, project “Molecular epidemiology of infectious diseases among children under five years in Thai Binh, Vietnam”). The study procedures were based on the good clinical practices recommended by the Declaration of Helsinki and its amendments. Informed signed consent was obtained from the legal guardians of children included in this study.

## Results

### Sociodemographic characteristics and housing conditions

A total of 467 patients with CAP were included. According to the guidelines of the Pediatric Infectious Diseases Society and the Infectious Diseases Society of America and the British Thoracic Society^2,10^, 128 (27%) cases were classified as severe pneumonia.

**Figure 1:**
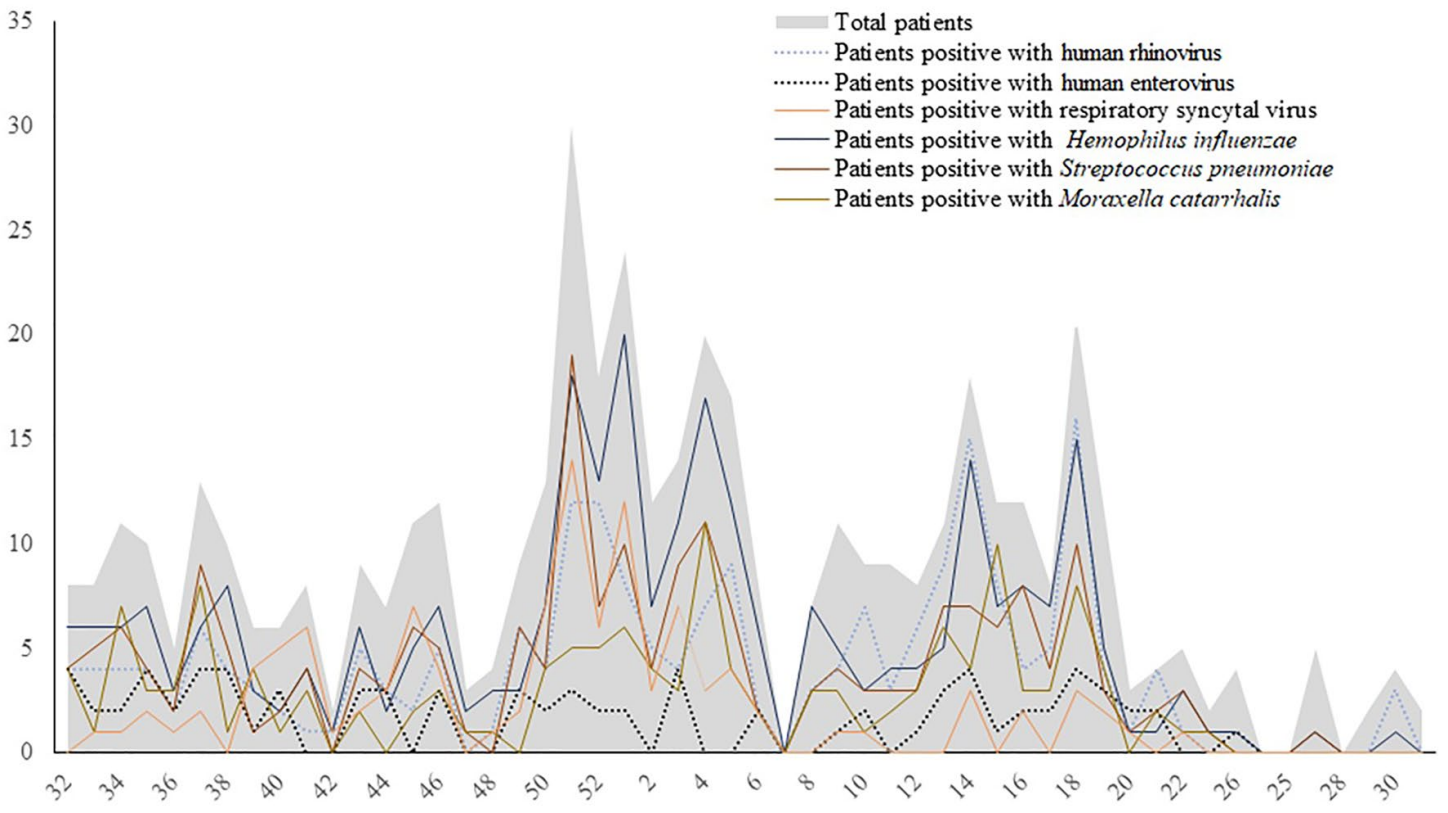
Number of patients and number of positive respiratory samples by week (2020–2021).

Higher numbers of patients were enrolled in December 2021–April 2022, corresponding roughly to the dry season in Thai Binh Province (Fig. 1). The mean age of participants was 15.4 months. The majority of patients were aged from 2 to 11 months (45.4%) and 12 to 23 months with the proportions (21.8%). Most of them were male (63%). One quarter lived with smokers at home, 16% mentioned that another household member was suffering from acute respiratory tract infections symptoms at the time of enrolment (Supplementary Table 2).

### Vaccination status, mode of admission and antibiotic treatment at enrolment

A majority of patients (89%) were fully vaccinated for their age according to the Vietnamese vaccination programme^8^. Vaccination rates against influenza and invasive pneumococcal diseases were low (7% and 9%, respectively). Only 6% of patients were transferred from district hospitals or other medical centres, and 43% had received antibiotic treatment before enrolment (Supplementary Table 2).

### Clinical features and routine laboratory investigation in the Thai Binh Paediatric Hospital

The most common symptoms were a cough (99%), tachypnoea (90%), rhinorrhoea (72%), wheezing (58%) and stridor when calm (40%). About 47% of patients were febrile. A proportion of 14% of ill children had oxygen saturation levels < 90% in ambient air. All patients had abnormal pulmonary auscultation (Table 1).

**Table 1:** Clinical and paraclinical features of the participants (N = 467).

Characteristics	Total (N = 467)	Non-severe pneumonia (N = 339)	Severe pneumonia (N = 128)	P-value
Symptoms and signs				
Fever	217 (46.5)	143 (42.2)	74 (57.8)	0.003
Cough	460 (98.5)	332 (97.9)	128 (100)	0.20
Tachypnoea	419 (89.7)	291 (85.8)	128 (100)	< 0.0001
Rhinorrhoea	336 (72.0)	227 (67.0)	109 (85.2)	< 0.0001
Wheezing	269 (57.6)	187 (55.2)	82 (64.1)	0.08
Stridor when calm	182 (40.0)	125 (36.9)	57 (44.5)	0.13
Chest indrawing	49 (10.5)	27 (8.0)	22 (17.2)	0.004
Grunting	105 (22.5)	–	105 (82.0)	–
Persistent vomiting	12 (2.6)	–	12 (9.4)	–
Inability to drink or breastfeed	6 (1.3)	–	6 (4.7)	–
Severe respiratory distress	4 (0.9)	–	4 (3.2)	–
Central cyanosis	3 (0.6)	–	3 (2.3)	–
Oxygen saturation < 90% in room air	66 (14.1)	–	66 (51.6)	–
Abnormal pulmonary auscultation	467 (100)	339 (100)	128 (100)	1.0
Chest X-rays, laboratory, and microbiological investigation in TBPH				
Chest X-ray				
Normal	6 (1.3)	4 (1.2)	2 (1.6)	0.55
Bronchial wall thickening	284 (60.8)	211 (62.2)	73 (57.0)	
Bilateral diffuse interstitial infiltrates	177 (37.9)	124 (36.6)	53 (41.4)	
White blood cell count (G/L)				
< 5	11 (2.3)	6 (1.8)	5 (3.9)	0.18
5–15	360 (77.1)	258 (76.1)	102 (79.7)	
> 15	96 (20.6)	75 (22.1)	21 (16.4)	
CRP levels (mg/dL)				
< 50	286 (61.2)	245 (72.3)	41 (32.0)	< 0.0001
≥ 50	181 (37.8)	94 (27.7)	87 (68.0)	
Positive blood culture	11/106 (10.4)	11/95 (11.6)	0/9 (0)	0.59
* Haemophilus influenzae*	1/11 (9.0)	1/11 (9.0)	–	–
* Streptococcus pneumoniae*	10/11 (91.0)	10/11 (91.0)	–	–
Positive for influenza Ag rapid test	3/100 (3.0)	0/68 (0)	3/32 (9.4)	0.03

Radiological findings showed that 38% of children had bilateral diffuse interstitial infiltrates. Three percent of patients had a white blood count < 5 G/L and 21% had a white blood count > 15 G/L, while 38% had CRP levels ≥ 50 mg/dL. Of the 106 patients tested for blood culture, 11 (10%) were positive, including ten for *S. pneumoniae* and one for *H. influenzae*. Of the 100 patients tested using the influenza Ag rapid test, 3% were positive (Table 1).

### Hospital treatment and outcome

Almost all patients (95%) were treated with antibiotics during their hospital stay, mostly with cephalosporins. The median duration of antibiotic treatment was seven days, ranging from 1 to 22 days. During hospitalisation, less than 1% required mechanical ventilation and 28% required supplemental oxygen. The median length of the hospital stay was 8 days, and 90% stayed 7 days or more. Most patients were discharged, but 3% were transferred to the National Paediatric Hospital and 3% were discharged against medical advice (Table 2). No deaths occurred.

**Table 2:** Treatment at hospital and outcome (N = 467).

Characteristics	Total (N = 467)	Non-severe pneumonia (N = 339)	Severe pneumonia (N = 128)	P-value
Empirical antimicrobial treatment	445 (95.3)	319 (99.7)	126 (98.4)	0.20
Beta-lactam	30 (6.4)	21 (6.2)	9 (7.0)	0.83
Cephalosporins	417 (89.3)	300 (88.5)	117 (91.4)	0.36
Aminoglycosides	79 (17.7)	54 (15.9)	25 (19.5)	0.35
Macrolides	14 (3.2)	13 (3.8)	1 (0.8)	0.13
Carbapenems	9 (2.2)	6 (1.8)	3 (2.3)	0.71
Glycopeptide antibiotics	3 (0.7)	1 (0.3)	2 (1.6)	0.18
Multiple antibiotics	118 (25.3)	82 (24.2)	36 (28.1)	0.38
Duration of antibiotic treatment in days, median (range)	7 (1–22)	7 (1–22)	7 (1–21)	0.21
Mechanical ventilation	2 (0.4)	0 (0)	2 (1.6)	0.08
Use of supplemental oxygen	132 (28.3)	4 (1.2)	128 (100)	< 0.0001
Length of stay in days, median (range)	8 (1–38)	8 (1–38)	8 (1–36)	0.16
Length of stay ≥ 7 days	419 (89.7)	303 (89.4)	116 (90.6)	0.69
Outcome				
Discharge	422 (94.4)	305 (95.6)	117 (91.4)	0.04
Transfer	11 (2.5)	4 (1.3)	7 (5.5)	
Discharge against medical advice	14 (3.1)	10 (3.1)	4 (3.1)	
Death	0 (0)	0 (0)	0 (0)	

### PCR results

**Figure 2:**
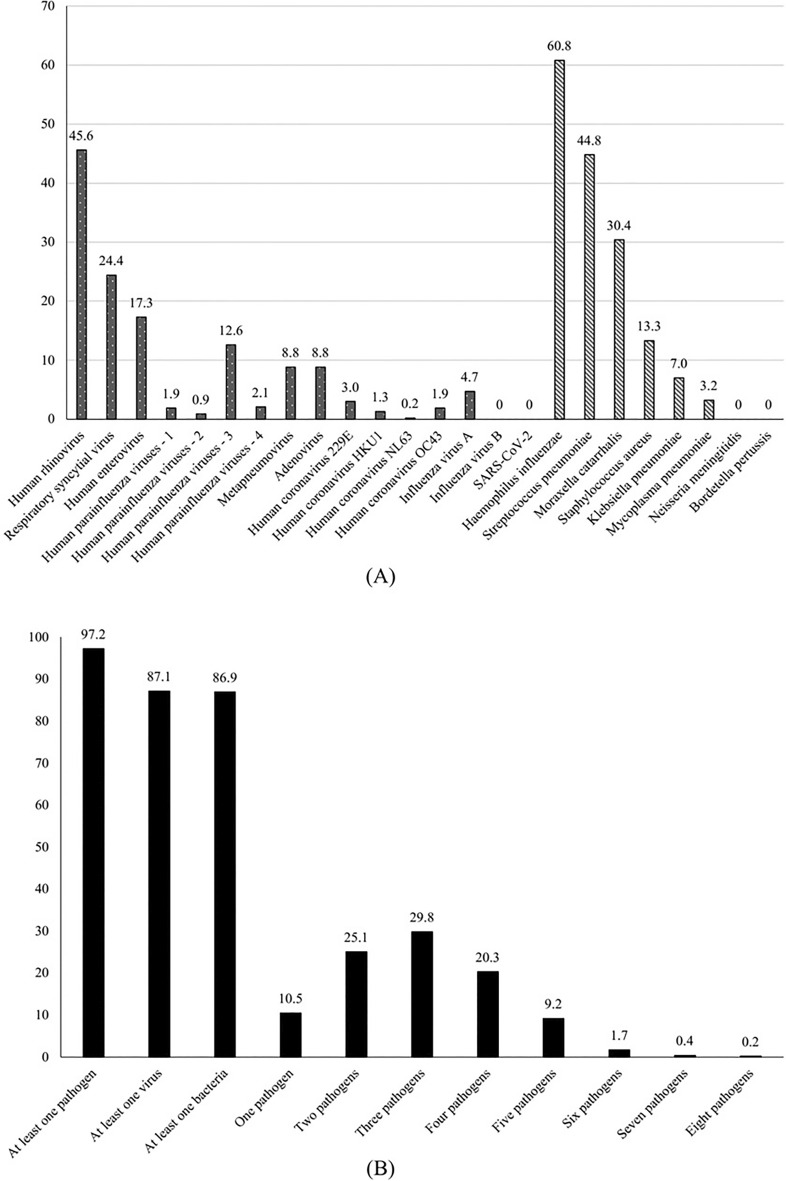
Results of real-time PCR performed on nasopharyngeal swabs among children with community-acquired pneumonia.

Almost all patients had a positive result (97%). Interestingly, the majority of patients (87%) were positive for multiple (up to eight) microorganisms (Fig. 2A and Supplementary Table 3).

Human rhinovirus (46%), respiratory syncytial virus (24%), human enterovirus (17%), and human parainfluenza viruses (17%) were the most frequent viruses. It was notable that no cases of SARS-CoV-2 infection were detected. *H. influenza* (61%), *S. pneumoniae* (45%) and *M. catarrhalis* (30%) were the most commonly found bacteria (Fig. 2B)*.*

According to age groups, most positive viral and bacterial microorganisms were detected in children aged from 2 to 11 months and 12 to 23 months (Supplementary Table 4).

The number of enterovirus-positive patients varied little over the time period of the study, while the majority of patients who were positive for rhinovirus, *H. influenzae* and *S. pneumoniae* were diagnosed in the winter and spring. Patients who were positive for respiratory syncytial virus were common in autumn and winter (Fig. 1).

### Factors associated with severe pneumonia among children

Table 3 shows the risk factors for severe community-acquired pneumonia among children by univariate and multivariate analysis.

**Table 3:** Risk factors for severe pneumonia among 467 children.

Risk factors	Non-severe pneumonia (N = 339)	Severe pneumonia (N = 128)	Univariate analysis		Multivariate analysis	
			OR (95% CI)	*P* value	OR (95% CI)	*P* value
Sociodemographic characteristics						
Age						
< 2 months	23 (6.8)	6 (4.7)	Ref			
2–11 months	152 (44.8)	60 (46.9)	1.51 [0.59–3.90]	0.39		
12–23 months	76 (22.4)	26 (20.3)	1.31 [0.48–3.57]	0.60		
24–35 months	58 (17.1)	25(19.5)	1.65 [0.60–4.55]	0.33		
36–59 months	30 (8.9)	11 (8.6)	0.41 [0.45–4.36]	0.56		
Male gender	215 (63.4)	80 (62.5)	0.96 [0.63–1.46]	0.85		
Vaccination status						
Fully vaccinated for age according to NEVP	308 (90.9)	109 (85.2)	0.58 [0.31–1.06]	0.08		
Vaccinated against seasonal influenza	20 (5.9)	13 (10.2)	1.80 [0.87–3.74]	0.11		
Vaccinated against IPD	28 (8.3)	13 (10.2)	1.26 [0.63–2.51]	0.52		
Familial factors						
Presence of smokers at home	65 (19.2)	42 (32.8)	2.06 [1.31–3.25]	0.002	2.11 [1.27–3.52]	0.004
Presence of persons with acute cough at home	46 (13.6)	28 (21.9)	1.78 [1.06–3.01]	0.03		
Laboratory findings						
WBC < 5 or > 15 G/L	81 (23.9)	26 (20.3)	0.81 [0.49–1.34]	0.41		
CRP levels ≥ 50 mg/dL	94 (27.7)	87 (68.0)	5.53 [3.56–8.60]	< 0.0001	6.11 [3.86–9.68]	< 0.0001
Chest X-ray with bilateral diffuse interstitial infiltrates	124 (36.6)	53 (41.4)	1.23 [0.81–1.86]	0.34		
PCR findings						
Human rhinovirus	166 (49.0)	47 (36.7)	0.60 [0.40–0.92]	0.02		
Respiratory syncytial virus	75 (22.1)	39 (30.5)	1.54 [0.98–2.43]	0.06	1.78 [1.07–2.96]	0.03
Human enterovirus	64 (18.9)	17 (13.3)	0.66 [0.37–1.17]	0.16		
Human parainfluenza virus	60 (17.7)	18 (14.1)	0.76 [0.43–1.35]	0.35		
Metapneumovirus	29 (8.6)	12 (9.4)	1.10 [0.55–2.24]	0.78		
Adenovirus	31 (9.1)	10 (7.8)	0.84 [0.40–1.77]	0.65		
Human coronaviruses	19 (5.6)	11 (8.6)	1.58 [0.73–3.43]	0.24		
Influenza A virus	14 (4.1)	8 (6.3)	1.55 [0.63–3.78]	0.34		
* Haemophilus influenzae*	196 (57.8)	88 (68.8)	1.60 [1.04–2.47]	0.03	1.66 [1.03–2.67]	0.04
* Streptococcus pneumoniae*	150 (44.3)	59 (46.1)	1.08 [0.72–1.62]	0.72		
* Moraxella catarrhalis*	108 (31.9)	34 (26.6)	0.77 [0.49–1.22]	0.27		
* Staphylococcus aureus*	49 (14.5)	13 (10.2)	0.67 [0.35–1.28]	0.22		
* Klebsiella pneumoniae*	20 (5.9)	13 (10.2)	1.80 [0.87–3.74]	0.11		
* Mycoplasma pneumoniae*	9 (2.7)	6 (4.7)	1.80 [0.63–5.17]	0.27		

The presence of smokers at home doubled the risk for severe pneumonia among children with aOR  2.11, 95% CI 1.27–3.52, *P* value = 0.004. CRP level ≥ 50 mg/dL was associated with a sixfold increased risk pf severe pneumonia (aOR  6.11, 95% CI  3.86–9.68, *P* value < 0.0001). Respiratory syncytial virus (aOR  1.78, 95% CI  1.07–2.96, *P* value = 0.03) and *H. influenzae* aOR  1.66, 95% CI  1.03–2.67, *P* value = 0.04) PCR detection were associated with approximately double the risk of severe disease (Table 3).

## Discussion

The primary pathogens causing CAP among children encompass a diverse range of typical bacteria, atypical bacteria, and viruses, as supported by previous studies^15–17^. Understanding the aetiology of CAP is crucial for effective diagnosis, treatment, and prevention strategies. In our particular study, we aimed to shed light on the prevalence and patterns of microorganisms involvement in paediatric CAP cases. Remarkably, our findings revealed a high prevalence rate of microorganisms detection by PCR in 97% of patients. This emphasises the potential impact of these microorganisms on the occurrence of CAP in the community.

What makes our study particularly intriguing is the discovery that a substantial proportion of patients, approximately 87%, exhibited co-infections with multiple microorganisms. This suggests a complex interplay between different agents, potentially contributing to the severity and complexity of the disease. In some cases, we observed up to eight different microorganisms coexisting within a single patient, suggesting the multifactorial nature of CAP. These co-infections involving multiple agents raise important questions about the mechanisms underlying their simultaneous presence and the potential synergistic effects they may have on disease progression^16,18^. Similarly, in a prospective descriptive study conducted among 141 Chinese children with severe CAP^19^, the authors showed that 92.2% of patients were positive for at least one microorganism by RT-PCR. In addition, 47.7% of patients were co-infected. The five most frequent bacteria species detected by PCR among children with pneumonia were *H. influenzae, S. pneumoniae, Methicillin-resistant S. aureus, M. catarrhalis* and *M. pneumoniae*^19,20^. Further research is needed to unravel the intricate interactions between these microorganisms and their role in shaping the clinical presentation and outcomes of paediatric CAP. Notably, studies involving healthy control patients might help to assess the pathogenic role of microorganisms in CAP symptoms. Moreover, identifying specific pathogens which are responsible for CAP in children is vital for appropriate and targeted treatment. Our study contributes to the growing body of evidence supporting the need for comprehensive diagnostic approaches that can detect multiple agents simultaneously. This is particularly important in cases of co-infections, where the use of broad-spectrum antibiotics alone may not be sufficient to effectively combat the diverse array of pathogens involved^2^.

Among the viral microorganisms detected, human rhinovirus accounted for the highest incidence at 46%. This finding highlights the substantial role played by rhinovirus in paediatric CAP cases. Additionally, respiratory syncytial virus (RSV) was responsible for 24% of the cases, making it another prominent viral pathogen associated with CAP in children. The study also identified human enterovirus (17%) and human parainfluenza viruses (17%) as significant contributors to the burden of disease. Understanding the viral aetiology of CAP in children is crucial for accurate diagnosis, appropriate management, and the development of effective preventive measures^2^. Indeed, these are viral agents for which there is no pharmaceutical prevention or specific treatment. Therefore, community infection prevention and monitoring of CAP patients caused by viruses is very important when it comes to reducing the burden of disease as well as the rate of inappropriate antibiotic use. The overuse of antibiotics in children with pneumonia has been reported not only in Thai Binh, Vietnam, but also in many other places around the world, especially in low-income countries.

In terms of bacterial agent, *H. influenzae, S. pneumoniae* and *M. catarrhalis* were the most frequently isolated. This finding aligns perfectly with previous research, which has consistently emphasised the significant role of these bacteria as causative agents of paediatric CAP^19,20^. The presence of these three bacterial agents underscores the complex microbial landscape involved in paediatric CAP. It is worth noting that while these bacteria accounted for a substantial proportion of cases, other pathogens, including viruses and atypical bacteria, may also contribute to the development of CAP in children^7^. Continued research efforts are essential to further comprehend the epidemiology, pathogenesis, and antibiotic susceptibility patterns of these bacterial agents in paediatric CAP. Understanding the prevalence and distribution of these bacteria, as well as the associated risk factors for the severity of disease and clinical outcomes will aid in the development of targeted prevention strategies, appropriate diagnostic approaches, and effective treatment regimens^21^.

Regarding risk factors for severe pneumonia among children, our study found that the presence of smokers in the family, CRP level ≥ 50 mg/dL, respiratory syncytial virus, and *H. influenzae* were associated with a risk of severe disease.

Previous studies have consistently shown that children exposed to household smoke are more vulnerable to respiratory infections, including pneumonia, compared to those living in smoke-free environments^3,22^. The link between household smoking and severe pneumonia in children has been quantified through various epidemiological studies. One such study, conducted on a large scale, revealed that the odds of severe pneumonia occurring in children exposed to household smoke were doubled compared to those from smoke-free homes^22^. Indeed, children admitted to the hospital for CAP from households where there were two or more smokers experienced an extended duration of hospitalisation and demonstrated a higher likelihood of needing intensive care, as compared to children from smoke-free households^22^. This finding underscores the detrimental impact of passive smoke on the respiratory health of children, highlighting the urgent need for effective tobacco control measures to protect vulnerable individuals.

Several studies have demonstrated a strong association between an increase in CRP levels and the risk of severe pneumonia in children, as shown in one meta-analysis^23^. Elevated CRP levels in pneumonia patients often indicate a more aggressive inflammatory response and a higher burden of infection. These children are more likely to experience severe respiratory distress, hypoxia, and other life-threatening complications. In previous studies, patients with high CRP levels were at a significantly higher risk of developing severe pneumonia, requiring admission to the intensive care unit (ICU), and experiencing longer hospital stays^24,25^. Another study by Masarweh et al*.* reported that elevated CRP levels were associated with a higher likelihood of developing pleural effusion, empyema, or necrotising pneumonia, all of which are indicators of severe disease^26^.

RSV is known as a leading cause of lower respiratory tract infections in young children^27^. RSV infection can range from mild respiratory symptoms to severe pneumonia, especially in vulnerable populations. Several studies have established a strong correlation between RSV infection and the increased severity of pneumonia in children. RSV-associated pneumonia often presents with rapid progression, increased respiratory distress, and a higher risk of complications, including the need for hospitalisation and intensive care^27^.

*H. influenzae* is a bacterial agent that commonly colonises children’s respiratory tracts. However, certain strains can cause invasive diseases such as pneumonia^28^. *H. influenzae* type B pneumonia is characterised by rapid onset, high fever, respiratory distress, and an increased likelihood of complications^28^. Timely diagnosis and treatment is essential to minimise the morbidity and mortality associated with *H. influenzae*-induced pneumonia. Several factors contribute towards the increased risk of severe pneumonia associated with *H. influenzae* infection^29^. Firstly, *H. influenzae* possesses various virulence factors that facilitate its colonisation and invasion of the respiratory tract. Secondly, children, especially those with underlying conditions or weakened immune systems, are particularly susceptible to *H. influenzae* pneumonia. Children attending day-care centres, living in crowded environments, or exposed to passive smoking are at higher risk of acquiring *H. influenzae* infections due to their increased exposure to respiratory secretions and compromised respiratory defences^30^.

Despite our efforts, this study has some limitations. Although RTIs cover a broad spectrum of diseases and severities, outpatients were not enrolled in this study. Consequently, the results are only representative of hospitalised patients. The study was conducted in a single hospital over a short period of time. The results only present a snapshot of aetiologies of hospital CAP in Vietnamese children. The highly sensitive nature of RT-PCR makes it possible to detect the remaining material of dead microorganisms together with active microbial agents. Some patients may have infections with viruses or bacteria which were not included in our study (including measles) or with pathogens yet to be discovered. The microorganisms found in the nasopharyngeal provide only indirect proof of the aetiologies of pneumonia and the detection of some pathogens by PCR might indicate respiratory carriage that does not necessarily account for the symptoms. However, a recent study showed a concordance in pathogen identification in the upper and lower respiratory tracts of children with pneumonia^19^. Another limitation of our study is the lack of a control group to assess the magnitude of healthy carriage of viruses and bacteria. In addition, because sampling technique was not standardised and because sampling was done at different stages of the disease depending on the time between onset of symptoms and children entry at hospital, we were not able to evaluate the viral or bacterial load of microorganisms detected by PCR as done by other authors^31^. Finally, due to responses to the second, third, and fourth waves of the COVID-19 pandemic in Vietnam, lockdowns and restriction measures were executed, which made it complicated to include patients and potentially affected the epidemiology of RTIs in Vietnam.

## Conclusion

Using a large panel of tested pathogens, our results identified the most frequent respiratory agents among children with pneumonia. Particularly, we found a high proportion of co-infections with multiple microorganisms. The presence of smokers in the family, RSV and *H. influenzae* infections were independent risk factors for severe disease. Case-controlled and metagenomic studies need to be performed to further investigate the role of the presence of microbial agents in the respiratory tract on pneumonia in children in order to differentiate between colonisation and infection.

### Supplementary Information

Supplementary Information.

## Data Availability

The datasets used and/or analysed during the current study are available from the corresponding author on reasonable request.
