# Supplementary material for: Viral and bacterial microorganisms in Vietnamese children with severe and non-severe pneumonia
Source: Sci Rep. 2024 Jan 2;14:120. doi: 10.1038/s41598-023-50657-5 (PMC10761988; doi:10.1038/s41598-023-50657-5)
Supplement: Supplementary file 1 — Supplementary Information. [file 41598_2023_50657_MOESM1_ESM.docx]

Supplementary Table 1. Classification of community-acquired pneumonia among children [1,2]

| **Clinical features of mild pneumonia** | **Clinical features of severe pneumonia** |
| --- | --- |
| Temperature <38.5 °C (101.3 °F) | Temperature ≥38.5 °C (101.3 °F) |
| Mild or absent respiratory distress:   - Increased respiratory rate (RR), but less than the age-specific RR that defines moderate to severe respiratory distress. - Mild or absent retractions. - No grunting. - No nasal flaring. - No apnoea. - Mild shortness of breath. | Moderate to severe respiratory distress:   - RR >70 breaths/minute for infants; RR >50 breaths/minute for older children - Moderate/severe suprasternal, intercostal, or subcostal retractions (<12 months) - Severe difficulty breathing (≥12 months) - Grunting - Nasal flaring - Apnoea - Significant shortness of breath |
| Normal colour | Cyanosis |
| Normal mental status | Altered mental status |
| Normoxaemia (oxygen saturation ≥92 percent in ambient air) | Hypoxemia (sustained oxygen saturation <90 percent in ambient air at sea level) |
| Normal feeding (infants); no vomiting | Not feeding (infants) or signs of dehydration (older children) |
| Normal heart rate | Tachycardia |
| Capillary refill <2 seconds | Capillary refill ≥2 seconds |

Supplementary Table 2. Sociodemographic data, familial factors, vaccination status and medical care before admission (N=467)

| **Characteristics** | **n (%)** |
| --- | --- |
| **Sociodemographic characteristics** |  |
| Age (mean ± 2SD, range) (months) | 15.4 ±13.3 (0-59) |
| Male gender | 295 (63.2) |
| Age group |  |
| < 2 months | 29 (6.2) |
| 2–11 months | 212 (45.4) |
| 12–23 months | 102 (21.8) |
| 24–35 months | 83 (17.8) |
| 36–59 months | 41 (8.8) |
| **Familial factors** |  |
| Smoker(s) at home | 107 (22.9) |
| Other household member with acute RTI at home | 74 (15.8) |
| **Vaccination status** |  |
| Fully vaccinated for their age according to NEVP | 417 (89.3) |
| Influenza during the past year | 33 (7.1) |
| IPD | 41 (8.8) |
| **Medical treatment before admission** |  |
| Patient transferred from district hospital/other medical centres | 30 (6.2) |
| Antibiotic use before hospitalisation | 201 (43.0) |
| Antibiotic self-prescription | 52/201 (25.8) |

Supplementary Table 3. Distribution of microorganisms among patients with co-infections

| Co-infections | n | % |
| --- | --- | --- |
| **Two microorganisms** |  |  |
| One virus - one bacteria | 87 | 18.6 |
| Two viruses | 15 | 3.2 |
| Two bacteria | 15 | 3.2 |
| **Three microorganisms** |  |  |
| One virus - two bacteria | 84 | 18.0 |
| Two viruses - one bacteria | 42 | 9.0 |
| Three viruses | 4 | 0.9 |
| Three bacteria | 9 | 1.9 |
| **Four microorganisms** |  |  |
| One virus - three bacteria | 37 | 7.9 |
| Two viruses - two bacteria | 43 | 9.2 |
| Three viruses - one bacteria | 12 | 2.6 |
| Four viruses | 1 | 0.2 |
| Four bacteria | 2 | 0.4 |
| **Five microorganisms** |  |  |
| One virus - four bacteria | 7 | 1.5 |
| Two viruses - three bacteria | 20 | 4.3 |
| Three viruses - two bacteria | 14 | 3.0 |
| Four viruses - one bacteria | 2 | 0.4 |
| Five viruses | 0 | 0.0 |
| **Six microorganisms** |  |  |
| Two viruses - four bacteria | 1 | 0.2 |
| Three viruses - three bacteria | 7 | 1.5 |
| Four viruses - two bacteria | 0 | 0.0 |
| Five viruses - one bacteria | 0 | 0.0 |
| **Seven microorganisms** |  |  |
| Three viruses - four bacteria | 1 | 0.2 |
| Four viruses - three bacteria | 1 | 0.2 |
| Five viruses - two bacteria | 0 | 0.0 |
| **Eight microorganisms** |  |  |
| Four viruses - four bacteria | 0 | 0.0 |
| Five viruses - three bacteria | 1 | 0.2 |
| **Total** | 405 | 86.7 |

Supplementary Table 4: Viral and bacterial organisms detected according to age groups.

| Microorganisms | Age group | | | | |
| --- | --- | --- | --- | --- | --- |
|  | < 2 months | 2–11 months | 12–23 months | 24–35 months | 36–59 months |
| Human rhinovirus  (N = 213) | 16  (7.5) | 102 (47.9) | 47  (22.1) | 31  (14.6) | 17  (9.4) |
| Respiratory syncytial virus  (N = 114) | 5  (4.4) | 52  (45.6) | 24  (21.1) | 25  (21.9) | 8  (7.0) |
| Human enterovirus  (N = 81) | 2  (2.4) | 34  (42.0) | 19  (23.5) | 19  (23.5) | 7  (8.6) |
| Human parainfluenza viruses  (N = 78) | 3  (3.8) | 32  (41.0) | 19  (24.4) | 18  (23.1) | 6  (7.7) |
| Metapneumovirus  (N = 41) | 2  (4.9) | 18  (43.9) | 9  (21.9) | 9  (21.9) | 3  (7.3) |
| Adenovirus  (N = 41) | 3  (7.3) | 15  (36.6) | 14  (34.2) | 6  (14.6) | 3  (7.3) |
| Human coronaviruses  (N = 30) | 0  (0) | 17  (56.7) | 4  (13.3) | 5  (16.7) | 4  (13.3) |
| Influenza virus A  (N = 22) | 1  (4.5) | 11  (50.0) | 4  (18.2) | 5  (22.8) | 1  (4.5) |
| *Haemophilus influenzae*  (N = 284) | 15  (5.3) | 112  (39.4) | 69  (24.3) | 63  (22.2) | 25  (8.8) |
| *Streptococcus pneumoniae*  (N = 209) | 9  (4.3) | 80  (38.3) | 56  (26.8) | 39  (18.6) | 25  (12.0) |
| *Moraxella catarrhalis*  (N = 142) | 5  (3.5) | 62  (43.7) | 31  (21.8) | 30  (21.1) | 14  (9.9) |
| *Staphylococcus aureus*  (N = 62) | 5  (8.1) | 30  (48.4) | 12  (19.4) | 6  (9.7) | 9  (14.4) |
| *Klebsiella pneumoniae*  (N = 33) | 4  (12.1) | 20  (60.6) | 5  (15.2) | 3  (9.1) | 1  (3.0) |
| *Mycoplasma pneumoniae*  (N = 15) | 1  (6.7) | 8  (53.4) | 2  (13.3) | 2  (13.3) | 2  (13.3) |

**References**

[1] Bradley JS, Byington CL, Shah SS, et al. The Management of Community-Acquired Pneumonia in Infants and Children Older Than 3 Months of Age: Clinical Practice Guidelines by the Pediatric Infectious Diseases Society and the Infectious Diseases Society of America. *Clin Infect Dis*. 2011;53(7):e25-e76. Doi:10.1093/cid/cir531

[2] Harris M, Clark J, Coote N, et al. British Thoracic Society guidelines for the management of community acquired pneumonia in children: update 2011. *Thorax*. 2011;66 Suppl 2:ii1-23. Doi:10.1136/thoraxjnl-2011-200598
